# Minimally invasive surgery for patients with advanced stage endometrial cancer

**DOI:** 10.7150/ijms.52293

**Published:** 2021-01-01

**Authors:** Sang Il Kim, Dong Choon Park, Sung Jong Lee, Ji Geun Yoo, Min Jong Song, Chan Joo Kim, Hae Nam Lee, Joo Hee Yoon

**Affiliations:** 1 Department of Obstetrics and Gynecology, St. Vincent's hospital, College of Medicine, The Catholic University of Korea, Seoul, Republic of Korea; 2 Department of Obstetrics and Gynecology, Seoul St. Mary's hospital, College of Medicine, The Catholic University of Korea, Seoul, Republic of Korea; 3 Department of Obstetrics and Gynecology, Daejeon St. Mary's hospital, College of Medicine, The Catholic University of Korea, Seoul, Republic of Korea; 4 Department of Obstetrics and Gynecology, Yeouido St. Mary's hospital, College of Medicine, The Catholic University of Korea, Seoul, Republic of Korea; 5 Department of Obstetrics and Gynecology, Uijeongbu St. Mary's hospital, College of Medicine, The Catholic University of Korea, Seoul, Republic of Korea; 6 Department of Obstetrics and Gynecology, Buchen St. Mary's hospital, College of Medicine, The Catholic University of Korea, Seoul, Republic of Korea

**Keywords:** endometrial cancer, advanced stage, minimally invasive surgery, adjuvant treatment, PORTEC-3

## Abstract

**Objective:** Compare the oncologic outcomes of patients with advanced stage endometrial cancer who were staged by minimally invasive surgery with the outcomes of patients who underwent open surgery.

**Methods:** Data from 138 patients with advanced stage endometrial cancer who were treated between January 2009 and January 2019 were reviewed. The patients' data were retrieved from five institutions. The patients were divided into two groups: those who underwent open surgery and those who underwent minimally invasive surgery. Tumor characteristics, recurrence rate, disease-free survival, and overall survival were compared according to surgical approach.

**Results:** Among the 138 patients included in this study, 72 underwent open surgery (52.2%) and 66 underwent MIS (47.8%). In patients with advanced-stage endometrial cancer, the recurrence rate was significantly higher among those who underwent open surgery (43.1% vs. 25.8%, p = 0.033). Patients with advanced-stage endometrial cancer who underwent open surgery had a significantly lower disease-free survival (p = 0.029) than those who underwent minimally invasive surgery, however, the overall survival (p = 0.051) was similar between the two groups.

**Conclusion:** Minimally invasive surgery showed better survival outcomes when compared to open surgery in advanced-stage EC patients irrespective of the histologic type.

## Introduction

In Korea, the incidence of endometrial cancer (EC) has been increasing 1, and it is now the most common gynecological cancer with an expected 3,261 new cases and 383 deaths in 2020 2.

Surgery is often the primary treatment for EC and involves total hysterectomy and bilateral salpingo-oophorectomy with lymph node assessment 3, 4. According to the National Comprehensive Cancer Network (NCCN), both open surgery and minimally invasive surgery (MIS) are acceptable treatment approaches for EC 4.

Unlike cervical and ovarian cancer, many randomized clinical trials showed similar oncologic outcomes of minimally invasive surgery (MIS) in EC compared to open surgery. And MIS showed lower rate of infection, transfusion, venous thromboembolism, and decreased hospital stay 5-12. However, most randomized studies were focused on patients with early stage and low-risk EC, and some retrospective studies reported that high-risk histologic subtype of EC is not a contraindication to MIS 13-16.

However, data reporting the survival outcomes and safety of MIS in advanced stage EC remains limited. For this reason, the European guidelines for the management of endometrial cancer considered that MIS can be considered in the management of advanced stage endometrial cancer while MIS is recommended in early stage endometrial cancer 17. This retrospective multicenter study aims to compare the oncologic outcomes of patients with advanced stage EC who were staged by MIS versus open surgery, irrespective of the histologic type.

## Material and Methods

This retrospective multicenter study was approved by the institutional review board of the Catholic University of Korea (Approval No. XC20RADI0115). The requirement of informed consent was waived due to the nature of the study. The study was conducted in accordance with the Declaration of Helsinki. From our institution's cancer registry, we identified patients who underwent primary surgical treatment for EC from January 2009 to January 2019. Data on 654 patients were recorded into a single database. Patients' data were retrieved from five institutions: Seoul St. Mary's hospital (n = 298), St. Vincent's hospital (n = 132), Yeouido St. Mary's hospital (n = 59), Uijeongbu St. Mary's hospital (n = 79), and Bucheon St. Mary's hospital (n = 85). Only patients who underwent primary surgery were eligible. Patients who refused adjuvant treatment, who received incomplete surgical staging, who had an early stage (stage I, II) EC or distant metastasis were excluded. Both pelvic and para-aortic lymphadenectomy were recommended. However, when pelvic lymph nodes were free of the disease, para-aortic lymphadenectomy could be omitted. The patients were segregated into two groups: those who underwent open surgery and those who underwent MIS. Robot-assisted surgery was included in the MIS group. After the surgery, adjuvant chemotherapy or radiotherapy was selectively implemented according to the disease stage and physician's discretion.

Overall survival (OS) was calculated from the date of initial diagnosis until cancer-related death or the last follow-up. Disease-free survival (DFS) was calculated from the date of initial diagnosis until the date of first disease progression or death or the date of last status if the patient was alive.

A subgroup analysis was performed by segregating patients based on the surgical approach. Differences in clinicopathologic characteristics were evaluated. We used the Student's *t*-test, chi-square test or Fisher's exact test for comparing variables. The Kaplan-Meier method with the log-rank test was used for comparing the survival outcomes between the two groups. All statistical analyses were performed using the SPSS statistical software (version 21.0, SPSS Inc., Chicago, IL, USA). We defined the threshold for statistical significance as *p* <0.05.

## Results

**Figure 1:**
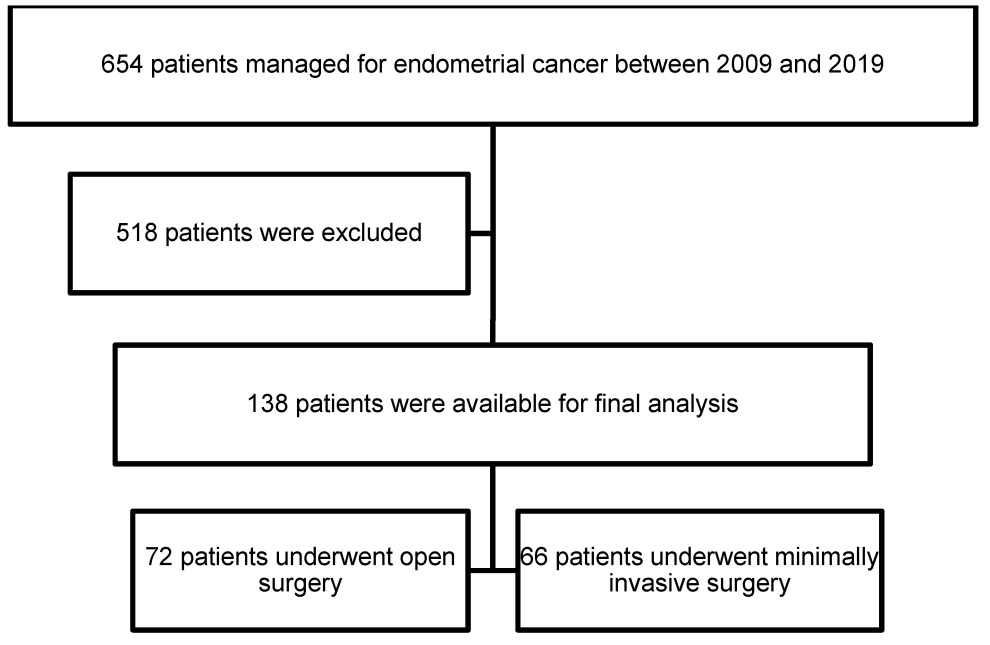
Flow chart

Overall, 138 patients met our inclusion criteria. Among the 138 patients who underwent primary surgery and had stage IIIA, IIIB, IIIC, and IVA disease, 72 underwent open surgery (52.2%) and 66 underwent MIS (47.8%). In the MIS group, 57 patients were scheduled for conventional laparoscopy (86.4%) and 9 for robotic surgery (13.6%). The patient selection flow chart is shown in Fig. 1.

The clinicopathologic characteristics of the patients are presented in Table 1. The mean age of the MIS group was 56.5 years, and the mean body mass index (BMI) was 24.7 kg/m^2^. The mean age of the open surgery group was 58 years, and the mean BMI was 24.9 kg/m^2^. No differences were observed between the two groups. In both the groups, approximately 70% of the patients had stage IIIC EC, and the two groups were comparable in terms of the stage, grade, deep myometrial invasion status, lymphovascular space invasion (LVSI) status, and lymphadenectomy status. Several characteristics were different between the two groups; patients who underwent open surgery significantly had larger tumors (median size, 6.1 vs 4.1 cm, *p* <0.0001), had a higher rate of cervical involvement (37.5% vs 21.2%, *p* = 0.042), and had more lymph nodes removed (median number, 38 vs 30, *p* = 0.014). All the patients received adjuvant treatment, and significantly more patients in the MIS group received combined adjuvant chemotherapy and radiotherapy.

During a median length of observation of 46 months (range: 3-127 months), 49 patients (35.5%) experienced disease recurrence (Table 2). Recurrences occurred in 31 (43.1%) of the 72 open surgery cases and 17 (25.8%) of the 66 MIS cases. Recurrence rate was significantly higher in the open surgery group (*p* = 0.033). In both the groups, most recurrences were found in stage IIIC EC: 26 (83.9%) of the 31 cases in the open surgery group and 13 (76.5%) of the 17 cases in the MIS group. In addition, the open surgery group showed significantly higher recurrence rate in stage IIIC patients (50.9% vs 28.3%, *p* = 0.037). Relapse location was not impacted by the surgical approach (*p* = 0.210). There were 30 (21.7%) cancer-related deaths in the entire cohort: 10 (15.2%) in the MIS group and 20 (27.8%) in the open surgery group (*p* = 0.098). The recurrence rate according to other factors, such as grade, tumor size, myometrial invasion depth, cervical involvement, LVSI, type of lymphadenectomy, and type of adjuvant treatment were not affected by the surgical route.

No differences were found between conventional laparoscopy and robotic surgery (Table 3).

**Figure 2:**
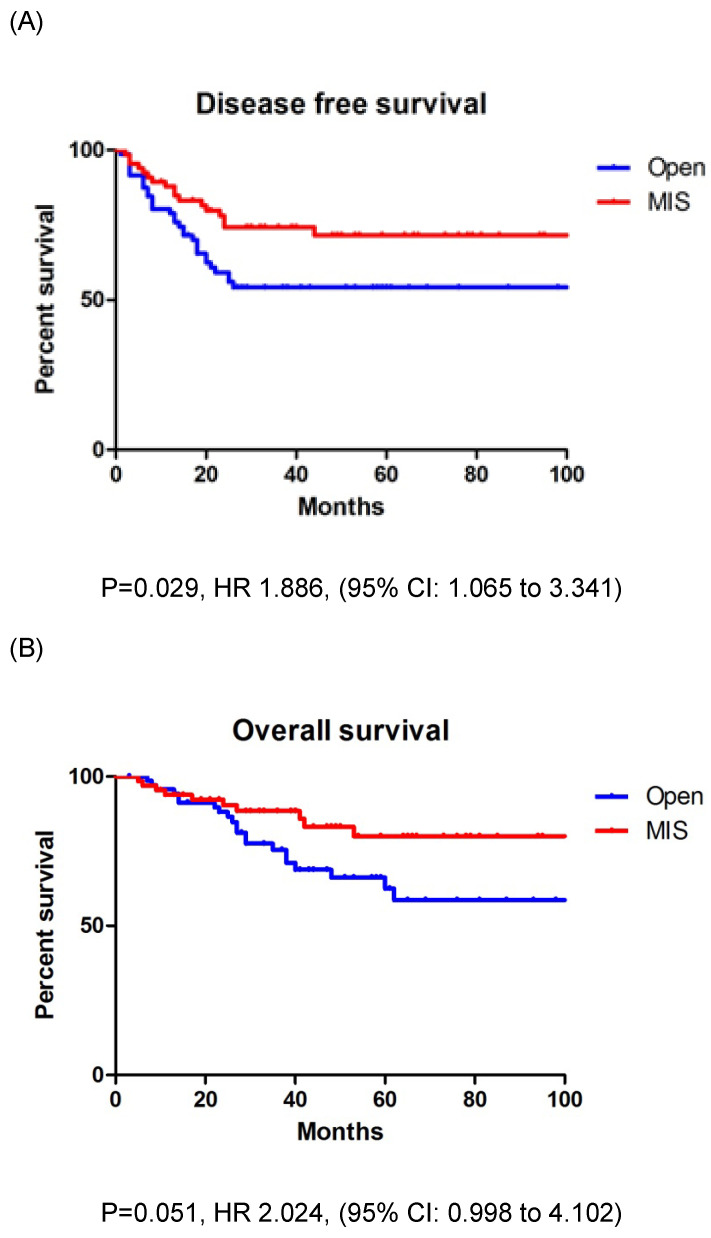
A: Disease free survival in entire cohort. B: Overall survival in entire cohort

**Figure 3:**
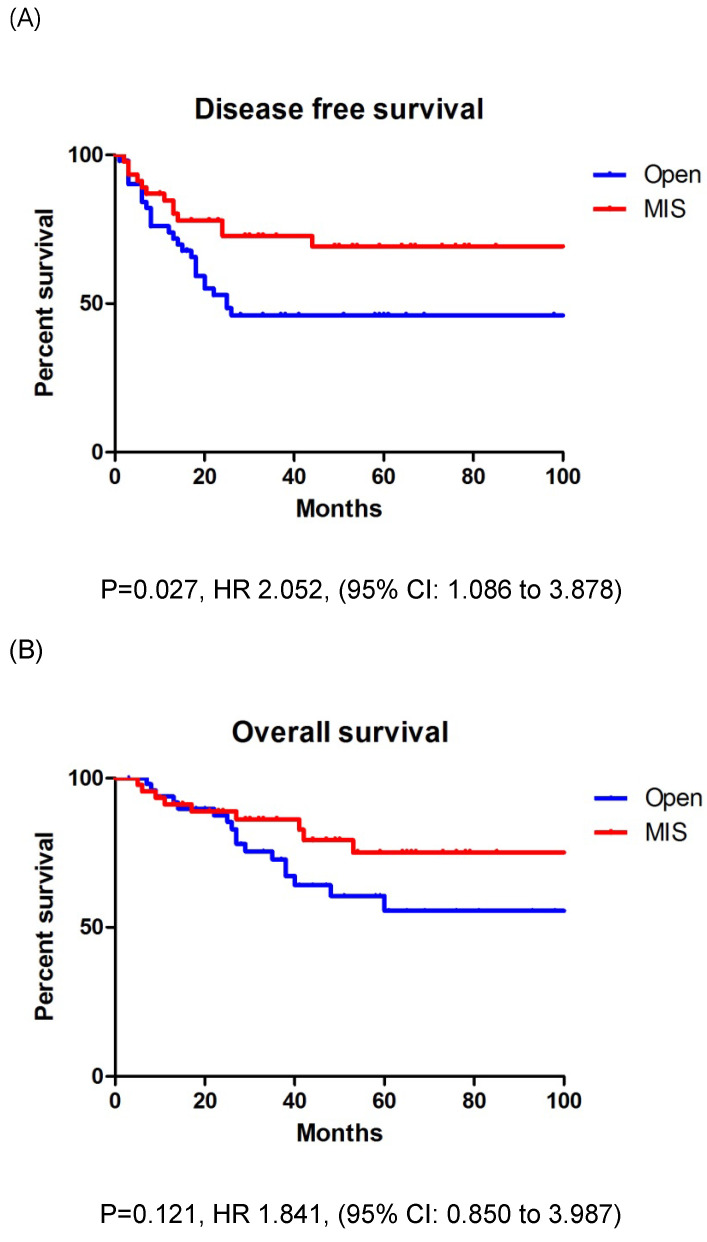
A: Disease free survival in stage IIIC patients. B: Overall survival in stage IIIC patients

In entire cohort, the patients who underwent open surgery had a significantly lower DFS (*p* = 0.029) than those who underwent MIS; however, the OS (*p* = 0.051) was similar between the two groups (Figs. 2A, B). The three-year DFS and OS rates were 54.2% and 68.8% in the open surgery group and 74.3% and 80.0% in the MIS group, respectively. The patients with stage IIIC EC who underwent open surgery had a significantly lower DFS (*p* = 0.027) than those who underwent MIS; however, the OS (*p* = 0.121) was similar between the two groups (Figs. 3A, B). The three-year DFS and OS rates were 46.1% and 67.2% in the open surgery group and 69.3% and 79.3% in the MIS group, respectively.

## Discussion

This study was performed for evaluating the oncologic safety of MIS in advanced stage EC patients. This retrospective study demonstrated that MIS is effective and better compared with open surgery for patients with advanced stage EC.

MIS for EC was first reported in 1992 18. Since that time, the volume of MIS compared to open surgery has gradually increased 19, and according to the current guideline, both MIS and open surgery are accepted as a treatment option for EC 4. Many randomized trials reported the safety of MIS for EC 5-12. MIS showed oncologic outcomes similar to open surgery but with fewer postoperative complications. However, most previously reported studies included patients with early stage and low-risk EC. Although a study by Tozzi *et al.* included advanced stage EC patients, the number of cases was too small: 6 patients with stage III disease. Some retrospective studies compared MIS with open surgery in high-risk EC patients and concluded that the high-risk histologic subtype was not a contraindication to MIS 13-16. However, even in those studies, the majority of patients had early stages I and II EC. Thus, in our study, we only included pathologically confirmed FIGO stage III and IV disease, and all histologic types were included. We found that histologic subtype does not affect the oncologic outcomes of patients undergoing MIS or open surgery for EC.

Fader *et al.* reported the largest retrospective multicenter study, which included 112 patients with advanced stage EC, and the patients who underwent MIS and open surgery showed similar survival outcomes 13. However, in contrast to their results, we observed a higher overall recurrence rate and lower DFS in the open surgery group compared with the MIS group. Similar results were reported in another study by Monterossi et al 16; this study compared patients with type II EC who underwent MIS and open surgery. A total of 283 patients were included, and 71 patients had stage III EC. Significant differences were observed in the recurrence rate with a higher recurrence rate and number in the open surgery group.

In our study, the rate of local and distant recurrence was similar in the two groups. This confirms that recurrence is not correlated with the surgical technique, which is consistent with results from previous studies; uterine manipulator seems to have no negative impact on the risk of local recurrence 20, 21.

In our study, para-aortic lymphadenectomy was more frequently performed in patients who underwent open surgery, and a significantly higher number of lymph nodes were removed in the open surgery group. However, significantly higher recurrence rate and lower DFS were found in patients with stage IIIC EC who underwent open surgery. The lymph node count and extent of para-aortic dissection did not appear to impact the recurrence rate and DFS in our analysis.

Tumor size >2 cm has been suggested to be relevant for predicting recurrence 22. In our study, the median tumor size was larger in patients who underwent open surgery, but the majority of patients had tumor size >2 cm, and the rate was similar between the two groups. Thus, the difference in tumor size is unlikely to influence the results.

In 2019, the results of a multicenter randomized phase 3 PORTEC-3 trial were reported 23. This trial investigated the benefit of combined adjuvant chemotherapy and radiotherapy versus pelvic radiotherapy alone for women with high-risk endometrial cancer. Among a total of 660 patients, 295 patients with stage III EC were included: 152 patients in the chemoradiotherapy group and 143 patients in the radiotherapy group. For women with stage III EC, a significant improvement in the OS and failure-free survival was observed in the chemoradiotherapy group. In our study, significantly more patients in the MIS group received chemoradiotherapy, and this could be related to the higher recurrence rate and lower DFS in the open surgery group. In addition, MIS is associated with lower complication rates and faster recovery after the surgery 13, 16. Thus, it would be advantageous to minimize the delay for adjuvant treatment, which could be related to the survival benefit in the MIS group.

Our study has several limitations. First, owing to the retrospective study design, inherent bias might exist. Second, the sample size might be insufficient to accurately compare the DFS and OS between the two groups. However, the sample size of our study was larger than that in other retrospective studies that included advanced stage EC. Third, variation in techniques, expertise, and outcomes among surgeons were not considered. Fourth, perioperative complications according to the surgical approach were not evaluated.

In conclusion, MIS showed better survival outcomes when compared to open surgery in advanced stage EC patients irrespective of the histologic type. Thus, MIS may be considered in patients with advanced stage, high-risk EC subtypes. After surgery, combined adjuvant chemotherapy and radiotherapy may be considered for advanced stage EC patients. Further well-designed, randomized studies are needed for evaluating the safety and feasibility of MIS in patients with advanced stage EC.

## Figures and Tables

**Table 1:** Clinopathological characteristics of patients

	Open (n = 72, %)	MIS (n = 66, %)	Total (n = 138, %)	P value
Age, mean	58.1	56.5	57.3	0.305
BMI (kg/m^2^), mean	24.88	24.66	24.78	0.741
FIGO stage				0.442
IIIA	16 (22.2)	16 (24.2)	32 (23.2)	
IIIB	2 (2.8)	4 (6.1)	6 (4.3)	
IIIC	51 (70.8)	46 (69.7)	97 (70.3)	
IVA	3 (4.2)	0	3 (2.2)	
Grade				0.365
Endometrioid 1	16 (22.2)	7 (10.6)	23 (16.7)	
2	20 (27.8)	37 (56.1)	57 (41.3)	
3	15 (20.8)	10 (15.1)	25 (18.1)	
Other high grade^*^	21 (29.2)	12 (18.2)	33 (23.9)	
Tumor size (cm), median	6.1	4.1	5.2	< 0.0001
Range	0.4 - 15	0.8 - 11.5	0.4 - 15	
< 2 cm	5 (6.9)	7 (10.6)	12 (8.7)	
≥ 2 cm	67 (73.1)	59 (89.4)	126 (91.3)	
Myometrial invasion ≥ 50%	55 (76.4)	42 (63.6)	97 (71.0)	0.136
Cervical involvement	27 (37.5)	14 (21.2)	41 (30.4)	0.042
LVSI positive	48 (66.7)	45 (68.2)	93 (68.1)	0.858
Lymphadenectomy				0.110
Pelvic	20 (27.8)	27 (40.9)	47 (34.1)	
Pelvic and para-aortic	52 (72.2)	39 (59.1)	91 (65.9)	
Number of LN removed				0.014
Median	38	30	35	
Range	6 - 101	3 - 79	3 - 101	
Adjuvant treatment				
Radiotherapy only	17 (23.6)	10 (15.2)	27 (19.6)	0.283
Chemotherapy only	30 (41.7)	21 (31.8)	51 (36.9)	0.289
Both	25 (34.7)	35 (53.0)	60 (43.5)	0.039

BMI, body mass index; FIGO, International Federation of Gynecology and Obstetrics; LVSI, lymphovascular space invasion; LN, lymph node. ^*^Serous, clear cell, carcinosarcoma, dedifferentiated, undifferentiated

**Table 2:** Oncologic outcomes of patients

	Open (n = 72, %)	MIS (n = 66, %)	Total (n = 138, %)	P value
Recurrences, total	31 (43.1)	17 (25.8)	48 (35.5)	0.033
Recurrences by FIGO stage				
IIIA	3/16 (18.8)	3/16 (18.8)	6/32 (18.8)	-
IIIB	1/2 (50.0)	1/4 (25.0)	2/6 (33.3)	-
IIIC	26/51 (50.9)	13/46 (28.3)	39/97 (40.2)	0.038
IVA	1/3 (33.3)	0	1/3 (33.3)	-
Recurrences by grade				
Endometrioid 1	3/16 (18.8)	1/7 (14.3)	4/23 (17.4)	0.795
2	8/20 (40.0)	9/37 (24.3)	17/57 (29.8)	0.240
3	6/15 (40.0)	1/10 (10.0)	7/25 (28.0)	0.179
Other high grade^*^	14/21 (66.7)	6/12 (50.0)	20/33 (60.6)	0.465
Recurrence site				0.210
Pelvic	5 (16.1)	5 (29.4)	10 (20.8)	
Extra-pelvic	12 (38.7)	7 (41.2)	19 (39.6)	
Both	14 (45.2)	5 (29.4)	19 (39.6)	
Median follow-up (months)	45.0	47.0	46.0	0.735
Range	3 - 120	5 - 127	3 - 127	
Death	20 (27.8)	10 (15.2)	30 (21.7)	0.098

FIGO, International Federation of Gynecology and Obstetrics. ^*^Serous, clear cell, carcinosarcoma, dedifferentiated, undifferentiated

**Table 3:** Robotic versus conventional laparoscopy

	Conventional (n = 57, %)	Robotic (n = 9, %)	P value
FIGO stage			0.389
IIIA	13 (22.8)	3 (33.3)	
IIIB	3 (5.3)	1 (11.1)	
IIIC	41 (71.9)	5 (55.6)	
Grade			0.608
Endometrioid 1	7 (12.3)	0	
2	31 (54.4)	6 (66.7)	
3	9 (15.8)	1 (11.1)	
Other high grade^*^	10 (17.5)	2 (22.2)	
Recurrences, total	14 (24.6)	3 (33.3)	0.684
Recurrences by FIGO stage			
IIIA	3/13 (23.1)	0/3	-
IIIB	0/3	1/1 (100)	-
IIIC	11/41 (26.8)	2/5 (40.0)	0.537
Recurrences by grade			
Endometrioid 1	1/7 (14.3)	0	-
2	8/31 (25.8)	1/6 (16.7)	0.228
3	1/9 (11.1)	0/1	-
Other high grade^*^	4/10 (40.0)	2/2 (100)	0.121
Median follow-up (months)	48.0	37.0	0.307
Range	5 - 127	19 - 73	
Death	9 (15.8)	1 (11.1)	0.716

FIGO, International Federation of Gynecology and Obstetrics. ^*^Serous, clear cell, carcinosarcoma, dedifferentiated, undifferentiated
